# Mental Health Status of North Korean Refugee Adolescents Living in South Korea: A Comparative Study with South Korean Adolescents

**DOI:** 10.3390/children12121689

**Published:** 2025-12-12

**Authors:** Susie Kim, Hyo-Seong Han, You-Shin Yi, Eun-Ju Bae, Youngil Lee, Chang-Min Lee, Ji-Yeon Shim, Dong-Sun Chung, Min-Sun Kim, Myung-Ho Lim

**Affiliations:** 1 Department of Counseling Psychology, Graduate School of Public Policy and Management, Dankook University, Cheonan 31116, Republic of Korea; 2 Department of Psychology, Graduate School, Dankook University, Cheonan 31116, Republic of Korea; 3 Department of Anatomy, College of Medicine, Dankook University, Cheonan 31116, Republic of Korea; 4 Department of Neurology, College of Medicine, Dankook University, Cheonan 31116, Republic of Korea; 5 Department of Nursing, Health and Medical School, Hanseo University, Seosan 31962, Republic of Korea; 6 W Psychiatric Clinics, Seongnam 13524, Republic of Korea; 7 Department of Psychology and Psychotheray, College of Health Science, Dankook University, Cheonan 31116, Republic of Korea

**Keywords:** refugees, adolescent, mental health, anxiety, depression, aggression

## Abstract

**Highlights:**

**What are the main findings?**

- North Korean refugee adolescents showed higher anxiety, depression, and somatic symptoms than general South Korean adolescents.
- North Korean refugee adolescents showed higher social immaturity, violation of rules, and aggressive behavior than general South Korean adolescents.

**What is the implication of the main finding?**

- North Korean refugee adolescents experience various psychological difficulties dur-ing their adaptation to South Korean society.
- These results can be used as basic data to detect mental health problems in North Korean adolescent refugees early and develop customized support plans.

**Abstract:**

The refugee population is increasing worldwide, and in South Korea, the refugee population, including children and adolescents, is also rapidly increasing. This study aimed to compare the psychological problems of North Korean refugee adolescents with those of South Korean adolescents and to evaluate their mental health characteristics. Methods: This cross-sectional comparative study assessed psychological problems using the Korean version of the Youth Self-Report Scale (K-YSR) among 206 South Korean adolescents and 130 North Korean refugee adolescents enrolled in middle and high schools in Gyeonggi Province. The inclusion criteria included adolescents aged 13–18 years at middle or high school and residing in South Korea for at least 6 months (for North Korean refugees). Data were collected in October 2025. Results: North Korean refugee adolescents showed significantly higher scores of anxiety/depression (*F* = 11.304, *p* < 0.001, *η*^2^ = 0.033), somatic symptoms (*F* = 20.997, *p* < 0.001, *η*^2^ = 0.060), social immaturity (*F* = 11.083, *p* < 0.001, *η*^2^ = 0.032), rule-breaking behavior (*F* = 12.851, *p* < 0.001, *η*^2^ = 0.037), and aggressive behavior (*F* = 50.386, *p* < 0.001, *η*^2^ = 0.132). Notably, the largest effect size (*η*^2^ = 0.132) was observed in the aggressive behavior domain, while the somatic symptoms also showed a moderate effect size (*η*^2^ = 0.060). In the ANCOVA analysis, controlling for gender and age as covariates, female students scored higher in the anxiety/depression and somatic symptoms domains, while male students scored higher in the rule-breaking behavior and aggressive behavior domains. Conclusions: North Korean refugee adolescents experience various psychological difficulties during their adaptation to South Korean society. These results can be used as basic data to detect mental health problems in North Korean adolescent refugees early and develop customized support plans.

## 1. Introduction

Around the world, the refugee population is increasing rapidly. According to the United Nations Refugee Agency (UNHCR), in 2023, the number of forced immigrants worldwide exceeded 110 million, of whom, children accounted for about 40% [1].

Refugee children and adolescents, particularly those from conflict zones, are exposed to severe mental health issues [2]. On the Korean Peninsula, divided since 1945, refugees from North Korea to South Korea have been increasing. The entry of North Korean refugees into South Korea has increased rapidly since the mid-1990s, and a total of 34,000 people had settled in South Korea as of the end of 2023 [3,4].

North Korean refugee children and adolescents are a vulnerable group in terms of mental health. In addition to political and economic difficulties, they are exposed to survival-threatening insecurity, family separation, unstable social situations, and the stress of adapting to a culturally heterogeneous environment. Children are more vulnerable to psychological stress than adults, and their longer life expectancy can prolong the adverse effects of stress.

Globally, the prevalence of mental illness among refugee children and adolescents is significantly higher than among non-refugee children and adolescents, with 22.7% reporting post-traumatic stress disorder (PTSD), 14.3% depression, and 17.1% anxiety disorders. In addition, refugee children were found to have various mental health problems such as attention deficit, behavioral problems, social withdrawal, sleep disorders, and learning disabilities [2,5,6].

In South Korea, a mental health study of 1618 North Korean refugee adolescents found that they had a 1.29 times higher odds ratio for psychiatric disorders compared to South Korean adolescents, including 2.33 times for post-traumatic stress disorder, 1.67 times for attention deficit hyperactivity disorder, 1.61 times for bipolar affective disorder, 1.53 times for major depressive disorder, and 1.25 times for anxiety disorders [7]. In addition, North Korean refugee adolescents have been reported to have higher rates of suicidal ideation, as well as depressive/anxiety symptoms, and relatively more psychiatric hospital visits [8,9].

North Korean refugee adolescents have unique characteristics that distinguish them from refugee adolescents in other countries. First, although they are of the same ethnic group, they experience adaptation stress due to cultural and social differences formed by division for over 80 years. In particular, there is a fear of bias and forced repatriation to North Korea due to long-term political and ideological education in North Korea. Second, they are exposed to complex stress factors such as trauma in the process of refuge and the experience of staying in a third country. Third, they have a dual burden of adapting to the competitive school education environment and rapid socioeconomic changes in South Korean society [10].

Research related to North Korean refugees has mainly focused on adult health problems, and comprehensive studies on the mental health of North Korean refugees, especially adolescents, are insufficient. In particular, studies compared to non-refugee South Korean children and adolescents using standardized measurement tools are very limited [11,12].

Children and adolescents are vulnerable to stress, and the adverse effects can be prolonged [13], which also negatively affects social adaptation [8,14].

Therefore, this study aimed to compare mental health problems such as anxiety/depression, somatic symptoms, social immaturity, rule violation, and aggressive behavior among North Korean refugees with those of South Korean adolescents using the Korean Youth Self-Report Scale (K-YSR), a standardized assessment tool.

## 2. Methods

### 2.1. Study Design

This study recruited participants through convenience sampling from middle and high schools in Gyeonggi-do. Participants were recruited from schools where North Korean refugee students were enrolled, and for the comparison group, general South Korean adolescents were recruited from the same schools. This study compared the mental health and psychological problems of 130 North Korean refugees and 206 South Korean adolescents at middle and high schools in Gyeonggi-do Province using the Korean version of the Youth Self-Report Scale (K-YSR). The inclusion criteria were as follows: (1) adolescents aged 13–18 years at middle or high school in Gyeonggi-do Province; (2) for North Korean refugee adolescents, residence in South Korea for at least 6 months; (3) ability to read and understand Korean. Exclusion criteria included (1) the presence of severe cognitive impairment or intellectual disability that would prevent completion of the self-report questionnaire and/or (2) acute psychiatric crisis requiring immediate intervention. This study was conducted as a self-report questionnaire, with the voluntary consent of all study participants and legal guardians, and the questionnaire took approximately 20–30 min to complete. Data were collected in October 2025.

### 2.2. Study Population

A total of 165 North Korean refugee adolescents were approached for study participation, of whom 150 provided consent. Of these, 130 completed the questionnaire. Reasons for non-participation included parental refusal (*n* = 10), student refusal (*n* = 5), and incomplete questionnaires (*n* = 20). For the general adolescent group, 230 students were approached, 220 provided consent, and 206 completed valid questionnaires. Reasons for non-participation were parental refusal (*n* = 8), student absence on the survey day (*n* = 10), and incomplete responses (*n* = 6). The study population consisted of 130 North Korean refugees currently residing in South Korea and 206 South Korean adolescents residing in the same region, with both groups of students enrolled in middle and high school. The participants were recruited with the cooperation of the teacher in charge, and after receiving the consent of the legal guardian and the participants themselves, the participants who met the selection criteria were finally selected.

### 2.3. Measurement

#### Korean Youth Behavior Assessment Scale (K-YSR)

This study used the Youth Behavior Rating Scale (YSR) developed by Achenbach and Rescorla [15] and standardized in Korean by Oh and Kim [16]. The YSR consists of 112 items and includes eight syndrome scales (anxiety/depression, withdrawal/depression, somatic symptoms, social immaturity, thinking problems, attention problems, rule breaking, and aggressive behavior) and a total score scale for internalizing, externalizing, and problem behavior. The reliability of the Korean version of the YSR ranged from 0.59 to 0.93, and the internal consistency reliability (Cronbach’s α) of each subscale in this study was 0.87 for anxiety/depression, 0.88 for withdrawal/depression, 0.89 for somatic symptoms, 0.88 for social immaturity, 0.88 for thought problems, 0.87 for attention problems, 0.89 for rule violations, 0.90 for rule breaking, and 0.88 for aggressive behavior. The Youth Self-Report has been translated and standardized in numerous countries worldwide, demonstrating cross-cultural validity. Moreover, the instrument features concise instructions and clearly described item content, making it accessible to adolescents from diverse backgrounds. Given these characteristics, the K-YSR was judged to be suitable for refugee adolescents in Korea. Regarding potential comprehension or administration issues, no significant difficulties were reported during data collection. Missing data were minimal and were judged not to influence the results.

### 2.4. Statistical Analysis

The collected data were analyzed using SPSS 25.0. Covariance analysis (ANCOVA) was performed in which age and gender were controlled as covariates to verify the difference in the K-YSR subscale scores of North Korean refugees and South Korean adolescents. The effect size was analyzed by calculating the partial *η*^2^. All statistical tests were performed as a two-sided test at the significance level of 0.05.

### 2.5. Ethics Statement

This study was conducted in accordance with the Declaration of Helsinki and was approved by the Research Ethics Committee of Dankook University (approval number DKU 2025-09-035). All participants and their legal guardians were informed of the purpose and procedures of the study, gave written informed consent, and were informed that they could withdraw their participation at any time during the study.

## 3. Results

### Demographic Characteristics

The demographic characteristics of the North Korean refugees and the South Korean adolescents are presented in Table 1.

The mean age of the South Korean adolescents was 15.85 years (SD = 0.358), and the mean age of the North Korean refugee adolescents was 16.81 years (SD = 1.469). The age difference between the groups was significant (t = −8.959, *p* < 0.001). There were 84 (40.8%) boys and 122 (59.2%) girls in the South Korean adolescent group, while there were 64 (49.2%) boys and 66 (50.8%) girls in the North Korean refugee adolescent group. A chi-square test showed no significant difference in the gender distribution between the two groups (χ^2^ = 2.31, *p* = 0.128).

In an analysis of covariance (ANCOVA), controlling for gender and age as covariates (Table 2), North Korean refugee adolescents had significantly higher levels of anxiety/depression (*F* = 11.304, *p* < 0.001, *η*^2^ = 0.033), somatic symptoms *(F* = 20.997, *p* < 0.001, *η*^2^ = 0. 060), social immaturity (*F* = 11.083, *p* < 0.001, *η*^2^ = 0.032), rule breaking (*F* = 12.851, *p* < 0.001, *η*^2^ = 0.037), and aggressive behavior (*F* = 50.386, *p* < 0.001, *η*^2^ = 0.132). In particular, the largest effect size (*η*^2^ = 0.132) was found in the aggressive behavior domain, while the somatic symptoms domain also showed a significant effect size (*η*^2^ = 0.060).

## 4. Discussion

North Korean refugees showed the largest effect size for aggressive behavior, followed by somatic symptoms, rule breaking anxiety/depression, and social immaturity This suggests that the trauma experienced in the process of attaining refuge is expressed as aggressive behavior, and North Korean refugees have difficulty controlling anger and aggression in the process of adapting to South Korean society. In this study, even after controlling for gender and age as covariates, differences between the groups were maintained, and these results show that the psychological problems of North Korean refugee adolescents are related to the stress experience of refuge and adaptation to South Korean society rather than demographic characteristics.

North Korean refugee adolescents showed a statistically significant difference in the area of externalizing problems. The largest effect size was found for aggressive behavior, and a significant effect size was found for rule violation. This suggests that North Korean refugee adolescents are having difficulty controlling their anger and controlling their behavior, and the trauma of the adaptation process is expressed as an external behavioral problem. These findings are consistent with previous studies by Hodes [17] and Mollica et al. [18], who found that refugee adolescents are at high risk for internalizing problems as well as behavioral problems.

Among internalizing problems, somatic symptoms had the most significant effect size, indicating a tendency for somatization, in which North Korean refugee adolescents express psychological stress as somatic symptoms. The anxiety/depression domain also showed a significant effect size, which is consistent with the findings of Hodes [17]. Mollica et al. [18] reported a significant increase in somatization symptoms in a study of Cambodian refugee adolescents, which may be related to the difficulty of directly expressing emotional distress in their sociocultural context.

In a study of 200 North Korean refugee adolescents, Baek et al. [19] identified factors associated with internalizing problems, including anxiety about health/aging, traumatic experiences, guilt, poor communication, school maladaptation, friendship problems, and family adaptation problems. A 10-year literature review also reported that the mental health problems of North Korean refugees were more closely related to the trauma of the refugee process and the stress of adapting to South Korean society than to age or gender.

Increased findings of depression/anxiety in refugee adolescents may be the result of the following mechanisms: (1) pre-migration/during-migration trauma including life-threatening escape experiences and family separation, (2) post-migration stressors encompassing acculturation challenges, educational gaps, and socioeconomic disadvantages, and (3) somatization processes reflecting cultural patterns of distress expression and limited access to mental health services. Notably, aggressive behavior showed the largest effect size, indicating this as a particularly pronounced area of difficulty and a distinctive characteristic of North Korean refugees, suggesting that the complex trauma they experienced manifests primarily through behavioral problems and somatic symptoms. Fazel et al. [20] identified both pre-migration trauma and post-migration stressors as key risk factors for psychological distress in displaced children resettled in high-income countries, consistent with the findings observed in our study.

The elevated externalizing problems, particularly the large effect size for aggressive behavior, are consistent with research documenting behavioral difficulties in refugee youth exposed to violence and instability. Lustig et al. [21] demonstrated that trauma exposure can manifest through externalizing symptoms in adolescent populations, with violence exposure normalizing aggressive responses through social learning processes. These findings help explain why aggression showed the most pronounced group difference in our study.

Significant differences in the area of social immaturity show that North Korean refugee adolescents have difficulty in peer relationships and adapting to school. This is consistent with the social dysfunction of refugee youth reported by Dangmann et al. [2]. The cultural and social differences caused by the division of North Korea and South Korea for over 80 years increase the burden of adaptation for North Korean refugee adolescents. North Korean refugees who speak the same language but are exposed to different social systems and values may have difficulty in social relationships along with identity confusion.

North Korean refugee adolescents have unique characteristics that distinguish them from refugee adolescents in other countries.

Although refugee children in general experience life threats, family separation, and trauma [2], North Korean refugee adolescents face a special situation in which they have to adapt to a culture that is of the same ethnic group but is heterogeneous.

They may have experienced traumatic events such as escape, unstable and poor living conditions in refugee camps, sometimes witnessing death, or experiencing torture and sexual violence themselves or in their families [22,23]. They are also often accompanied by developmental damage, such as loss of attachment objects, deprivation of educational opportunities [24], and are more likely to live with family members who exhibit depression and anxiety even in resettlement [25].

The disparity between political ideology education in North Korea and the liberal democratic system in South Korea can cause great confusion in adolescence, when self-identity is being formed. These ideological and cultural differences have a compounding effect on the psychological adaptation of North Korean refugees.

North Korean refugees may have relatively less cultural stress than other countries, because South Korea shares the same language as North Korea and has a friendly social atmosphere [26]. However, more than 80 years have passed since the division in 1945, and there is a large gap in ideology and culture between North and South Korea. In particular, North Korean refugee adolescents who are self-conscious may suffer great confusion due to different ideological and cultural differences. Therefore, it is necessary to understand the sociocultural background of North Korean refugee adolescents and to take an approach that considers stress factors in-depth and is multifaceted [7].

This study suggests that North Korean refugee adolescents may have more emotional problems than European refugee adolescents attending school [26]. Portes and Rumbaut [27] reported that refugee families have more mental health problems than families in general, and children in refugee families have more physical and psychological problems than children in general families, due to frequent domestic violence, parent conflicts, and family conflicts.

This study has several limitations. First, the use of self-report questionnaires limited the scope of objective assessment. Future research should include comprehensive studies incorporating not only student self-report questionnaires but also parent and teacher evaluations to enhance inter-rater reliability. Second, the cross-sectional research design meant that the study was a one-time assessment; so, follow-up assessments should be conducted. Longitudinal studies are needed to examine the long-term effects on North Korean refugees. Third, this study drew samples from selected schools in a specific region and, therefore, may not fully represent all North Korean refugee adolescents nationwide, which limits the generalizability of the findings. We would like to see future studies that include regional schools across the country, utilize a wider variety of assessment tools, and conduct long-term follow-up assessments. Fourth, an age difference was observed between groups. Although age was controlled as a covariate, this difference appears to reflect migration-related educational disruptions and may influence mental health outcomes through differences in developmental maturity, coping skills, and peer relationships. In addition, we were unable to control for several covariates (length of stay in South Korea, family situation, trauma exposure, socioeconomic status, etc.) other than age that could influence immigration.

Despite the limitations, this study is a meaningful study that objectively and systematically compared the mental health problems of North Korean refugees and South Korean adolescents using the K-YSR, a standardized assessment tool in South Korea.

We found that North Korean refugees have high levels of both internalizing and externalizing problems and that they have the highest difficulties with aggressive behavior and somatic symptoms.

Future research should include longitudinal studies with larger samples and comprehensive studies that include out-of-school refugees. Research should also be conducted to develop effective intervention programs and validate their effectiveness.

## 5. Conclusions

This study confirms that North Korean refugee adolescents have significantly higher levels of both internalizing and externalizing problems compared to South Korean peers. These findings demonstrate the complex psychological difficulties that refugee adolescents face during the adaptation process.

This study provides important implications for the development of customized intervention programs for North Korean refugees. Considering that the largest effect size was aggressive behavior, intensive interventions for anger control and behavioral management are required first. In addition, high scores on somatic symptoms show a tendency to physically express psychological pain; so, an understanding of somatization symptoms and an appropriate therapeutic approach are needed.

Difficulties in social immaturity suggest the need for programs to improve peer relationships and social skills.

## Figures and Tables

**Table 1:** Demographic Characteristics of South Korean adolescents and North Korean refugee adolescents.

Variable	Category	South Korean Adolescents (*n* = 206)	North Korean Refugee Adolescents (*n* = 130)	Total (*n* = 336)	Statistics	*p*-Value
Age						
	Mean ± SD	15.85 ± 0.358	16.81 ± 1.469	-	*t* = −8.959	<0.001 ***
Gender						
	Male	84 (40.8%)	64 (49.2%)	148 (44.0%)	*χ*^2^ = 2.311	0.128
	Female	122 (59.2%)	66 (50.8%)	188 (56.0%)		

*** *p* < 0.001. Notes: Age differences between groups were analyzed by the independent samples *t*-test, and gender distribution differences were analyzed by the chi-square test.

**Table 2:** Comparison of psychological problems between the South Korean adolescents and North Korean refugee adolescents.

Psychological Problem Domain	South Korean Adolescents	North Korean Refugee Adolescents	F-Value	*p*-Value	Partial *η*^2^
Anxious/Depressed	53.04	56.14	11.304	0.001 ***	0.033
Withdrawn/Depressed	54.92	55.98	1.151	0.284	0.003
Somatic Complaints	53.30	57.52	20.997	0.000 ***	0.060
Social Immaturity	52.30	55.08	11.083	0.001 ***	0.032
Thought Problems	53.89	54.05	0.033	0.855	0.000
Attention Problems	53.38	53.23	0.034	0.855	0.000
Rule-Breaking Behavior	51.28	53.56	12.851	0.000 ***	0.037
Aggressive Behavior	50.70	55.79	50.386	0.000 ***	0.132

Note: Adjusted mean values with gender and age controlled as covariates. *** *p* < 0.001. Effect sizes: small (0.01–0.059), medium (0.06–0.139), large (≥0.14).

## Data Availability

The datasets generated during and/or analyzed during the current study are available from the corresponding author on reasonable request.

## References

[1] UNHCR (2023). Mid-Year Trends 2023.

[2] Dangmann C., Dybdahl R., Solberg Ø. (2022). Mental health in refugee children. Curr. Opin. Psychol..

[3] Ministry of Unification (2025). Status of North Korean Defectors Policy.

[4] Ministry of Unification (2020). 2020 Unification White Paper.

[5] Blackmore R., Gray K.M., Boyle J.A., Fazel M., Ranasinha S., Fitzgerald G., Misso M., Gibson-Helm M. (2020). Systematic Review and Meta-analysis: The Prevalence of Mental Illness in Child and Adolescent Refugees and Asylum Seekers. J. Am. Acad. Child Adolesc. Psychiatry.

[6] Bamford J., Fletcher M., Leavey G. (2021). Mental Health Outcomes of Unaccompanied Refugee Minors: A Rapid Review of Recent Research. Curr. Psychiatry Rep..

[7] Lee R., Lee S.M., Hong M., Oh I.-H. (2025). Psychiatric Health Risks in North Korean Refugee Youths Resettled in South Korea. JAMA Netw. Open.

[8] Lee Y.M., Shin O.J., Lim M.H. (2012). The Psychological Problems of North Korean Adolescent Refugees Living in South Korea. Psychiatry Investig..

[9] Choi S.K., Min S.J., Cho M.S., Joung H., Park S.M. (2011). Anxiety and Depression among North Korean Young Defectors in South Korea and Their Association with Health-Related Quality of Life. Yonsei Med. J..

[10] (2013). National Knowledge Information System (NKIS). Education Data Portal. https://www.nkis.re.kr/subject_view1.do?otpId=KEDI00024962&otpSeq=0.

[11] Lee Y., Lee M., Park S. (2017). Mental Health Status of North Korean Refugees in South Korea and Risk and Protective Factors: A 10-Year Review of the Literature. Eur. J. Psychotraumatol..

[12] Lee R., Lee S.M., Hong M., Oh I.-H. (2022). Assessing Mental Illness Risk Among North Korean Refugees and Immigrants Resettled in South Korea. JAMA Netw. Open.

[13] Park J., Catani C., Hermenau K., Elbert T. (2019). Exposure to Family and Organized Violence and Associated Mental Health in North Korean Refugee Youth Compared to South Korean Youth. Confl. Health.

[14] Noh J.-W., Lee S.H. (2020). Trauma History and Mental Health of North Korean Defectors. Curr. Behav. Neurosci. Rep..

[15] Achenbach T.M. (2001). Manual for ASEBA School-Age Forms & Profiles.

[16] Kim M.Y., Kim Y.A., Oh K.J. (2012). Standardization Study for the Korean Version of the Teacher’s Report Form. Korean J. Sch. Psychol..

[17] Hodes M. (2000). Psychologically Distressed Refugee Children in the United Kingdom. Child Psychol. Psychiatry Rev..

[18] Mollica R.F., Poole C., Son L., Murray C.C., Tor S. (1997). Effects of War Trauma on Cambodian Refugee Adolescents’ Functional Health and Mental Health Status. J. Am. Acad. Child Adolesc. Psychiatry.

[19] Baek H.J., Kil E.B., Yoon I.J., Lee Y.L. (2006). A Study on the Countermeasure for North Korean Adolescent Refugees I: Focusing on the Adaptation to the South Korean Society.

[20] Jeon W.T. (2000). For Man’s Unification.

[21] Fazel M., Reed R.V., Panter-Brick C., Stein A. (2012). Mental Health of Displaced and Refugee Children Resettled in High-Income Countries: Risk and Protective Factors. Lancet.

[22] Lustig S.L., Kia-Keating M., Knight W.G., Geltman P., Ellis H., Kinzie J.D., Keane T., Saxe G.N. (2004). Review of Child and Adolescent Refugee Mental Health. J. Am. Acad. Child Adolesc. Psychiatry.

[23] Jeon W., Hong C., Lee C., Kim D.K., Han M., Min S. (2005). Correlation between Traumatic Events and Posttraumatic Stress Disorder among North Korean Defectors in South Korea. J. Trauma. Stress.

[24] Venta A., Cuervo M. (2022). Attachment, Loss, and Related Challenges in Migration. Curr. Opin. Psychol..

[25] Kang H.R. (2007). A Study of Psychosocial Adjustment of North Korean Refugee Adolescents in South Korea: Focused on Depression and Anxiety.

[26] Fazel M., Wheeler J., Danesh J. (2005). Prevalence of Serious Mental Disorder in 7000 Refugees Resettled in Western Countries: A Systematic Review. Lancet.

[27] Portes A., Rumbaut R.G. (2001). Legacies: The Story of the Immigrant Second Generation.

